# A Cross-Sectional Study on Mental Health Problems of Medical and Nonmedical Students in Shandong During the COVID-19 Epidemic Recovery Period

**DOI:** 10.3389/fpsyt.2021.680202

**Published:** 2021-06-11

**Authors:** Xiaolei Zheng, Yuji Guo, Hui Yang, Liyan Luo, Bailiu Ya, Hong Xu, Zhiwei Xue, Qing Li, Jiale Shi, Jianzhong Bi, Wen Ma, Ping Wang

**Affiliations:** ^1^Department of Neurology, The Second Hospital, Cheeloo College of Medicine, Shandong University, Jinan, China; ^2^Department of Histology and Embryology, Cheeloo College of Medicine, Shandong University, Jinan, China; ^3^Department of Epidemiology, School of Public Health, Cheeloo College of Medicine, Shandong University, Jinan, China; ^4^Department of Physiology, School of Basic Medical, Jining Medical University, Jining, China; ^5^Department of Philosophy School of Marxism, Dezhou University, Dezhou, China; ^6^Department of Clinical Medicine School, Cheeloo College of Medicine, Shandong University, Jinan, China; ^7^Department of Clinical Medicine, The Second Hospital, Cheeloo College of Medicine, Shandong University, Jinan, China; ^8^Department of Basic Medical School, Cheeloo College of Medicine, Shandong University, Jinan, China; ^9^Center for Clinical Neurolinguistics, School of Foreign Languages and Literature, Shandong University, Jinan, China

**Keywords:** COVID-19, nonmedical college students, PHQ-9, ISI, mental health problems

## Abstract

**Background:** The coronavirus disease 2019 (COVID-19) pandemic has resulted in a plethora of psychological problems worldwide since its onset in December 2019. In the upheaval period, compared with medical college students, nonmedical students' psychological state deserves additional concern due to their lack of medical knowledge. Although the epidemic in China has been largely controlled for several months, the mental health problems resulting from the COVID-19 epidemic persist to this day. In this study, we assessed the mental health problems and associated risk factors experienced by nonmedical vs. medical college students in universities of Shandong Province during the COVID-19 epidemic recovery period.

**Methods:** An online survey was conducted over the period from 17 to 19 December 2020. A total of 954 Chinese college students (486 nonmedical and 468 medical students) from three universities of Shandong Province participated in the survey. Mental health variables were assessed with use of Patient Health Questionnaire-9 (PHQ-9), Generalized Anxiety Disorder-7 (GAD-7), and Insomnia Severity Index (ISI).

**Results:** Compared with medical students, nonmedical college students had higher prevalence rates of depression (53.9 vs. 46.4%; *p* = 0.020) and insomnia (28.0 vs. 22.4%, *p* = 0.049), as well as higher total scores on the PHQ-9 (*p* = 0.03) and ISI (*p* < 0.01). Among nonmedical college students, being female and native of non-Shandong were risk factors for anxiety and depression (*p* < 0.01), while only native of non-Shandong for insomnia (*p* < 0.01). Among medical students, age (*p* < 0.01) and living in rural areas (*p* = 0.04) were risk factors for depression, while only age (*p* < 0.05) was a risk factor for anxiety and insomnia.

**Conclusion:** Nonmedical college students in the universities of Shandong Province had more mental health problems and more risk factors for developing them during the COVID-19 epidemic recovery period than medical students. These nonmedical students require additional attention and recovery programs to alleviate the increased incidence of psychological problems related to COVID-19.

## Introduction

The novel coronavirus disease 2019 (COVID-19) outbreak was first detected and reported in Wuhan, China in late December 2019 (1). Since being declared a global health emergency by the World Health Organization (WHO), the COVID-19 epidemic has spread worldwide resulting in massive disruptions to everyday life (2). According to the WHO, as of 3:02 p.m. CET on 5 December 2020, there were 65,257,767 confirmed global cases of COVID-19, including 1,513,179 deaths (3). Unfortunately, no effective treatments were available for this pandemic (4, 5), and in the absence of widespread vaccination, containment of COVID-19 was problematic, resulting in immeasurable disasters and losses throughout the world.

Increasing evidences have indicated that people were experiencing psychological problems caused by stress in response to negative information on COVID-19, changes in daily routines, and the uncertainty regarding the future of the epidemic (6). The psychological impact of COVID-19, which has been reported within the general public (7), healthcare workers (8), and elderly adults (9), was characterized by increases in anxiety, depression, and insomnia. Inevitably, as a special social group, college students' daily routines changed dramatically in a short period after the COVID-19 epidemic began, as they had to leave campus, adjust to social distancing, and adapt to online learning platforms. Thus, increases in the incidence of mental health problems experienced by college students have been reported. During the height of the COVID-19 epidemic in China, anxiety of college students was reported as being relatively high, a condition that was augmented in those having acquaintances or relatives infected with COVID-19 (10). With the global pandemic of COVID-19, a study of 8,004 college students in France (11) and another of 162 in America (12) showed that students suffered from particularly high levels of anxiety and depression, which were associated with over-focusing on the information of COVID-19, difficulties in academic activities, and employment losses. Taken together, there is no doubt that the COVID-19 epidemic has exerted a substantial psychological impact among college students on a global scale.

College students' psychological problems can significantly impair their academic success and health behaviors (13). Notably, these mental health problems related to the COVID-19 epidemic are not going away soon, and may be heightened and persist over time, thus affecting future careers and personal opportunities. Previous studies have revealed that the psychological problems of students whose routine life were significantly affected during a prior infectious disease epidemic persist to this day (14, 15). Therefore, the mental health of college students needs long-term attention in all periods of the COVID-19 epidemic, including the early period, the epidemic recovery period, and even the post-COVID-19 era.

Interestingly, compared with nonmedical students, medical students have deeper medical knowledge of COVID-19 and professional training (16). They deal with the epidemic with different mentalities and then are affected psychologically differently. Thus, an important topic of investigation involves comparing the mental health effects of the COVID-19 epidemic within medical vs. nonmedical students. Such a study would assist in gaining insights into the psychological mechanisms of stress during epidemics and constructing an effective psychological support system for different population groups. As summarized above, the present research focuses on the mental health status of medical and nonmedical students in the universities of Shandong Province during the COVID-19 epidemic recovery period, then measures the levels of anxiety, depression, and insomnia of students via an online survey, and further explores the risk factors for them.

## Materials and Methods

### Design, Procedure, and Participants

This was a cross-sectional study performed via an online survey conducted over the period from 17 to 19 December of 2020, 11 months after the COVID-19 epidemic outbreak founded in Wuhan (17). This period of investigation corresponded to the recovery stage after the outbreak of COVID-19 in China (18), i.e., the COVID-19 epidemic recovery period.

From the 16 cities in Shandong Province, three cities were selected at random (Jinan, Dezhou, and Jining), and then one University from each of these three cities was randomly selected for this survey. The selected universities consisted of Shandong University, Jining Medical University, and Dezhou University. In order to facilitate a unified management, all grades in the selected universities possessed their own WeChat groups, which included all undergraduate and postgraduate students. All the participants in the survey came from those WeChat groups, and none was infected by COVID-19.

The applet with a questionnaire was sent to the WeChat group, informing each of the students that they were welcome to participate in the online survey. Before responses, all students were informed of the purpose of the survey, aiming to better understand the mental health states of college students associated with the COVID-19 epidemic. A simple math question (i.e., 14–5=?) was included at the end to ensure the quality and completeness of responses with the questionnaire. Participants failing to complete the survey received a warning on unanswered questions from the online applet. The online applet did not give warnings to those who gave up. As a result, the participants were those who volunteered to take the online survey and completed all questions of the questionnaire. This study was approved by the Medical Ethics Committee of the Second Hospital of Shandong University [No. KYLL-2020(LW)-064].

### Measurements

Demographic data garnered from the questionnaire included sex, age, major (medical students, i.e., students of medicine, vs. nonmedical students), education status (undergraduate vs. postgraduate), native place (Shandong vs. non-Shandong), living areas (urban vs. rural), household income per year (≤ 100,000, 100,000–200,000, and ≥200,000 RMB), and risk of contact with COVID-19 patients in community, i.e., as being an infected individual who lives in the same community as the participants in the survey, whether or not they wear a mask or maintain an adequate social distance. Participants were also asked whether they have had insomnia or psychiatric disorders prior to COVID-19 and whether they were having organic diseases [the question was “Do you currently have any organic disease? (diagnosed by medical examination in the hospital)”], and those who replied positively were automatically excluded by the applet. In addition, insomnia, anxiety, and depression symptoms as experienced in students after the onset of COVID-19 were assessed.

Symptoms of depression were assessed via the Patient Health Questionnaire-9 (PHQ-9) (19), a self-report questionnaire with a nine-item scale widely used in China (20). The items are scored on a four-point scale ranging from 0 to 3 with participants' ratings reflecting the depression experienced over the preceding 2 weeks (0—not at all, 1—several days, 2—more than half of the days over this 2-week period, and 3—nearly every day). Previous studies suggest that the total score ranges from 0 to 27 (0–4: without depression symptoms, 5–9: with mild depression symptoms, 10–14: with moderate depression symptoms, 15–19: with moderate to severe depression symptoms, and 20–27: with severe depression symptoms) (21).

Symptoms of anxiety were assessed via the Generalized Anxiety Disorder Scale (GAD-7) (22); the reliability and validity of this scale have been well-established (23, 24). GAD-7 is a self-report questionnaire with a seven-item scale. The items are scored on a four-point scale ranging from 0 to 3 with participants' ratings reflecting the anxiety experienced over the preceding 2 weeks (0—not at all, 1—several days, 2—more than half of the days over this 2-week period, and 3—nearly every day). The total score ranges from 0 to 21 (0–4: without anxiety symptoms, 5–9: with mild anxiety symptoms, 10–13: with moderate anxiety symptoms, 14–18: with moderate to severe anxiety symptoms, and 19–21: with severe anxiety symptoms) (22).

Symptoms of insomnia were assessed via the Insomnia Severity Index (ISI), a seven-item self-report index assessing the severity of initial, middle, and late insomnia, sleep problem interference with daily functioning, satisfaction with current sleep pattern, noticeability of impairment caused by the sleep problem, and worry about sleep problems (25). The items are scored on a five-point scale ranging from 0 to 4 (26), with participants rating their sleep-related experiences over the preceding 2 weeks. The total score ranges from 0 to 28 (0–7: without insomnia, 8–14: with mild insomnia, 15–21: with moderate insomnia, and 22–28: with severe insomnia).

### Statistical Analyses

Data analyses were performed via the IBM SPSS Statistical Software (version 19). χ^2^ tests were used to compare group differences of categorical variables. Mann–Whitney tests were used to compare independent groups on continuous variables nonnormally distributed. Multivariate logistic regression analyses were performed using stepwise variable selection, and all variables were entered into the model to explore independent influence for different risk dimensions, such as insomnia, anxiety, and depression. Subgroup analyses were performed for medical and nonmedical college students. All hypotheses were tested at a significance level of 0.05.

## Results

A total of 954 college students completed the survey as presented via WeChat in December 2020. Table 1 presented sociodemographic features of the whole sample and compared 468 medical students (440 undergraduate and 28 postgraduate) to 486 nonmedical students (437 undergraduate and 49 postgraduate). Study participants were predominantly female (61.6%) and native of Shandong (71.2%). Only 9.6% of participants reported a risk of contact with COVID-19 patients in the community.

**Table 1:** Sociodemographic characteristics in medical college students vs. nonmedical college students.

**Characteristics**	**Total**	**Medical college students**	**Nonmedical college students**	***P*-value**
	**(*n* = 954)**	**(*n* = 468)**	**(*n* = 486)**	
**Age (year)**	21.1 ± 1.2 (18–28)	21.5 ± 1.3 (18–28)	20.9 ± 1.5 (18–25)	0.005
**Gender**, ***n*** **(%)**				0.468
Male	366 (38.4%)	185 (39.5%)	181 (37.2%)	
Female	588 (61.6%)	283 (60.5%)	305 (62.8%)	
**Education**, ***n*** **(%)**				0.020
Undergraduate	877 (91.9%)	440 (94.0%)	437 (89.9%)	
Postgraduate	77 (8.1%)	28 (6.0%)	49 (10.1%)	
**Shandong native**, ***n*** **(%)**				0.897
No	275 (28.8%)	134 (28.6%)	141 (29.0%)	
Yes	679 (71.2%)	334 (71.4%)	345 (71.0%)	
**Risk of contact with COVID-19 patients in community**, ***n*** **(%)**				0.260
No	862 (90.4%)	428 (91.5%)	434 (89.3%)	
Yes	92 (9.6%)	40 (8.5%)	52 (10.7%)	
**Household income per year**, ***n*** **(%)**				0.034
≤ 100,000 RMB	526 (55.1%)	264 (56.4%)	262 (53.9%)	
100,000–200,000 RMB	314 (32.9%)	161 (34.4%)	153 (31.5%)	
≥200,000 RMB	114 (12.0%)	43 (9.2%)	71 (14.6%)	
**Living areas**, ***n*** **(%)**				0.245
Urban	305 (32.0%)	158 (33.8%)	147 (30.3%)	
Rural	649 (68.0%)	310 (66.2%)	339 (69.7%)	

*COVID-19, the coronavirus disease 2019. χ^2^ tests for group differences of categorical variables; Mann–Whitney tests for independent groups on continuous variables*.

As shown in Table 2, nonmedical students showed higher prevalence rates of depression (53.9 vs. 46.4%; *p* = 0.020) and insomnia (28.0 vs. 22.4%, *p* = 0.049) than medical students, but there was no significant difference in the prevalence rates of anxiety between nonmedical and medical students (36.4 vs. 32.7%; *p* = 0.226). The majority of college students reporting depression, anxiety, or insomnia rated their experiences as being mild or moderate. More specifically, for nonmedical and medical students, the mild/moderate prevalence of depression was 30.2/14.4% and 25.7/11.3%, anxiety was 26.5/4.1% and 21.4/6.2%, and insomnia was 21.6/5.8% and 19.4/2.6%, respectively. There were no statistically significant differences between nonmedical and medical students for the severity of depression (*p* = 0.586), anxiety (*p* = 0.238), or insomnia (*p* = 0.062).

**Table 2:** Mental health status in medical college students vs. nonmedical college students.

**Characteristics**	**Total**	**Medical college students**	**Nonmedical college students**	***P*-value**
	**(*n* = 954)**	**(*n* = 468)**	**(*n* = 486)**	
**PHQ-9 (Depression)**, ***n*** **(%)**				0.020
0–4 (asymptomatic)	475 (49.8%)	251 (53.6%)	224 (46.1%)	
5–27 (symptomatic)	479 (50.2%)	217 (46.4%)	262 (53.9%)	
***Stage***				0.586
5–9 (mild)	267 (28.0%)	120 (25.7%)	147 (30.2%)	
10–14 (moderate)	123 (12.9%)	53 (11.3%)	70 (14.4%)	
15–19 (moderate to severe)	66 (6.9%)	29 (6.2%)	37 (7.6%)	
20–27 (severe)	23 (2.4%)	15 (3.2%)	8 (1.7%)	
**GAD-7 (Anxiety)**, ***n*** **(%)**				0.226
0–4 (asymptomatic)	624 (65.4%)	315 (67.3%)	309 (63.6%)	
5–21 (symptomatic)	330 (34.6%)	153 (32.7%)	177 (36.4%)	
***Stage***				0.238
5–9 (mild)	229 (24.0%)	100 (21.4%)	129 (26.5%)	
10–13 (moderate)	49 (5.1%)	29 (6.2%)	20 (4.1%)	
14–18 (moderate to severe)	37 (3.9%)	18 (3.8%)	19 (3.9%)	
19–21 (severe)	15 (1.6%)	6 (1.3%)	9 (1.9%)	
**ISI (Insomnia)**, ***n*** **(%)**				0.049
0–7 (asymptomatic)	713 (74.7%)	363 (77.6%)	350 (72.0%)	
8–28 (symptomatic)	241 (25.3%)	105 (22.4%)	136 (28.0%)	
***Stage***				0.062
8–14 (mild)	196 (20.6%)	91 (19.4%)	105 (21.6%)	
15–21 (moderate)	40 (4.2%)	12 (2.6%)	28 (5.8%)	
22–28 (severe)	5 (0.5%)	2 (0.4%)	3 (0.6%)	

*PHQ-9, Patient Health Questionnaire-9; GAD-7, Generalized Anxiety Disorder-7; ISI, Insomnia Severity Index. χ^2^ tests for group differences of categorical variables; Mann–Whitney tests for independent groups on continuous variables*.

As indicated in Table 3, nonmedical students also showed higher total scores of PHQ-9 (*p* = 0.03) and ISI (*p* < 0.01) than medical students, but there was no significant difference in the total scores of GAD-7 between nonmedical and medical students (*p* = 0.07). Compared with medical students, nonmedical students showed higher scores on three of the nine items on the depressive symptom scale, and four of the seven items on the insomnia symptoms scale, including items 3 (early awakening; *p* = 0.04), 4 (satisfaction; *p* < 0.01), 5 (interference; *p* < 0.01), and 6 (noticeable; *p* = 0.02). No significant difference in anxiety levels between the two groups was present (Table 3).

**Table 3:** Psychological manifestations of medical college students vs. nonmedical college students.

**Characteristics**	**Total**	**Medical college students**	**Nonmedical college students**	***P-*value**
	**(*n* = 954)**	**(*n* = 468)**	**(*n* = 486)**	
**PHQ-9 total score**	5.83 ± 5.68	5.55 ± 5.78	6.10 ± 5.57	0.03
Item 1: Little interest in doing things	0.74 ± 0.81	0.72 ± 0.83	0.76 ± 0.80	0.24
Item 2: Feeling down/depressed/hopeless	0.68 ± 0.79	0.67 ± 0.81	0.70 ± 0.78	0.33
Item 3: Sleep disorder, or sleeping too much	0.69 ± 0.86	0.66 ± 0.83	0.73 ± 0.89	0.32
Item 4: Feeling tired or lack of energy	0.84 ± 0.85	0.81 ± 0.88	0.86 ± 0.82	0.04
Item 5: Loss of appetite or eating too much	0.70 ± 0.90	0.67 ± 0.89	0.72 ± 0.90	0.26
Item 6: Feel bad, or feel like a failure	0.63 ± 0.84	0.59 ± 0.83	0.68 ± 0.85	0.04
Item 7: Hard to focus	0.90 ± 0.95	0.82 ± 0.91	0.98 ± 0.97	<0.01
Item 8: Moving slowly, or fidgeting or fidgeting	0.42 ± 0.73	0.40 ± 0.71	0.44 ± 0.74	0.30
Item 9: The idea of dying or hurting oneself	0.23 ± 0.58	0.22 ± 0.57	0.23 ± 0.58	0.60
**GAD-7 total score**	3.76 ± 4.63	3.60 ± 4.66	3.91 ± 4.59	0.07
Item 1: Feeling nervous/anxious/on edge	0.69 ± 0.79	0.66 ± 0.78	0.72 ± 0.79	0.20
Item 2: Not being able to stop worrying	0.57 ± 0.80	0.56 ± 0.81	0.59 ± 0.80	0.46
Item 3: Worry too much about all sorts of things	0.67 ± 0.87	0.61 ± 0.84	0.73 ± 0.90	0.04
Item 4: Hard to relax	0.54 ± 0.78	0.53 ± 0.77	0.56 ± 0.79	0.41
Item 5: Fidgeting with restlessness	0.40 ± 0.70	0.40 ± 0.72	0.39 ± 0.69	0.69
Item 6: Become easily annoyed or irritable	0.53 ± 0.76	0.49 ± 0.75	0.57 ± 0.76	0.05
Item 7: Feeling scared of something terrible	0.36 ± 0.68	0.35 ± 0.70	0.36 ± 0.66	0.49
**ISI total score**	4.87 ± 4.88	4.49 ± 4.73	5.24 ± 4.50	<0.01
Item 1: Falling asleep	0.58 ± 0.82	0.56 ± 0.79	0.61 ± 0.84	0.62
Item 2: Staying asleep	0.39 ± 0.71	0.37 ± 0.68	0.40 ± 0.74	0.88
Item 3: Early awakening	0.38 ± 0.69	0.33 ± 0.61	0.43 ± 0.76	0.04
Item 4: Satisfaction	1.21 ± 1.09	1.12 ± 1.08	1.30 ± 1.09	<0.01
Item 5: Interfere	0.99 ± 0.99	0.89 ± 0.99	1.07 ± 0.99	<0.01
Item 6: Noticeable	0.70 ± 0.91	0.63 ± 0.86	0.77 ± 0.95	0.02
Item 7: Worried	0.62 ± 0.86	0.59 ± 0.84	0.65 ± 0.88	0.29

*PHQ-9, Patient Health Questionnaire-9; GAD-7, Generalized Anxiety Disorder-7; ISI, Insomnia Severity Index. Mann–Whitney test for independent samples*.

The multivariate logistic regression analyses (Table 4) showed that being female (odds ratio [OR], 1.83; 95% confidence interval [CI], 1.24–2.70; *p* < 0.01) and native of non-Shandong (OR, 2.13; 95% CI, 1.40–3.25; *p* < 0.01) were risk factors for depression among nonmedical college students, while age (OR, 1.85; 95% CI, 1.35–2.52; *p* < 0.01) and living in rural areas (OR, 1.51; 95% CI, 1.01–2.24; *p* = 0.04) were risk factors for the medical college students. Two variables were independently associated with anxiety risk factors among nonmedical college students: being female (OR, 1.53; 95% CI, 1.01–2.30; *p* = 0.04) and native of non-Shandong (OR, 2.22; 95% CI, 1.48–3.35; *p* < 0.01), while only age (OR, 1.70; 95% CI, 1.26–2.30; *p* < 0.01) was a risk factor for the medical college students. For insomnia symptoms, native of non-Shandong (OR, 1.84; 95% CI, 1.96–2.83; *p* < 0.01) was selected as an independent risk factor among nonmedical students, while age (OR, 1.40; 95% CI, 1.02–1.91; *p* = 0.04) as a risk factor was found for medical college students.

**Table 4:** Outcomes of mental health manifestations.

**Variables**	**OR (95%CI)**	***P-*value**
**MODELS FOR PHQ**		
***MCSs***		
Age (year)	1.85 (1.35, 2.52)	<0.01
Education (postgraduate vs. undergraduate)	0.04 (0.01, 0.20)	<0.01
Living areas (rural vs. urban)	1.54 (1.03, 2.30)	0.04
***NMCSs***		
Sex (female vs. male)	1.83 (1.24, 2.70)	<0.01
Native of Shandong (no vs. yes)	2.13 (1.40, 3.25)	<0.01
***Total students***		
Sex (female vs. male)	1.36 (1.04, 1.78)	0.03
Native of Shandong (no vs. yes)	1.59 (1.19, 2.12)	<0.01
Living areas (rural vs. urban)	1.47 (1.10, 1.94)	<0.01
Risk of contact with COVID-19 patients in community (yes vs. no)	1.65 (1.05, 2.59)	0.03
**MODELS FOR GAD**		
***MCSs***		
Age (year)	1.70 (1.26, 2.30)	<0.01
Education (postgraduate vs. undergraduate)	0.02 (0.00, 0.17)	<0.01
***NMCSs***		
Sex (female vs. male)	1.53 (1.01, 2.30)	0.04
Native of Shandong (no vs. yes)	2.22 (1.48, 3.35)	<0.01
***Total students***		
Education (postgraduate vs. undergraduate)	0.33 (0.14, 077)	0.01
Native of Shandong (no vs. yes)	1.45 (1.08, 1.95)	0.01
**MODELS FOR ISI**		
***MCSs***		
Age (year)	1.40 (1.02, 1.91)	0.04
Education (postgraduate vs. undergraduate)	0.11 (0.02, 0.80)	0.03
***NMCSs***		
Native of Shandong (no vs. yes)	1.86 (1.21, 2.86)	<0.01
***Total students***		
Native of Shandong (no vs. yes)	1.40 (1.01, 1.92)	0.04

*OR, odds ratio; CI, confidence interval; MCSs, medical college students; NMCSs, nonmedical college students; COVID-19, the coronavirus disease 2019. Multivariate logistic regression analyses using stepwise variable selection*.

## Discussion

Previous studies have suggested that public health emergencies can exert significant effects on the mental health of college students (27). An online survey during the maximal period of the COVID-19 epidemic reported that, when asked to learn online at home, nonmedical college students showed higher levels of anxiety and depression than medical students (28). However, limited information is available on the COVID-19 epidemic impact on the mental health of nonmedical college students during the COVID-19 epidemic recovery period, when the epidemic was largely contained and students returned to campus to continue normal academic activities for several months. The present study is the first to investigate the levels of depression, anxiety, and insomnia of both medical and nonmedical students in universities of Shandong during the COVID-19 epidemic recovery period, as well as the risk factors for them. The findings of this study can serve as the foundation for the development of more effective protocols for use in protecting college students' physical and mental health from the stress of the COVID-19 epidemic.

Our survey results revealed for the first time that the prevalence of depression and insomnia among nonmedical students was higher than that of medical students in universities of Shandong in December 2020 when assessed during the COVID-19 epidemic recovery period. Interestingly, no statistically significant differences in the severity of depression and insomnia were obtained between these nonmedical and medical students. It seems likely that the increase of psychological distress experienced by nonmedical students may be associated with their relative lack of medical knowledge regarding COVID-19. As the global epidemic continued to intensify, they were increasingly concerned about the domestic re-emergence of the epidemic, resulting in an over-focus on negative information. In contrast, the more comprehensive knowledge resulting from their biomedical backgrounds enables medical students to have more confidence in the containment of the epidemic and take more aggressive precautions (16, 29). Recent researches showed that students with a higher awareness of COVID-19 experienced lower risks of mild anxiety, suggesting that the improved cognition of the epidemic was beneficial for the mental health (30). These findings were similar to those as reported for the SARS epidemic, where knowledge of the etiology, prevention, and treatment of the infectious disease aided in alleviating students' anxiety about its outbreak (31, 32). Encouragingly, with the training on the Novel Coronavirus Infection Pneumonia Protection, Diagnosis and Treatment Plan for all medical staff combined with adequate supplies of medical protections, no more doctors have been infected with COVID-19 among ~40,000 medical personnel nationwide supporting Hubei medical services (33). Therefore, we suggest that, to improve the mental health problems of nonmedical students, relevant government departments and universities should develop specialized education for them to improve their cognition of COVID-19 transmission, treatment, prognosis, and prevention.

An additional possibility to consider is that the higher levels of depression and insomnia in nonmedical students may be amplified by their pre-existing immediate responses during the maximal period of the COVID-19 epidemic. Xie et al. (28) reported that in the maximal period of the epidemic, the immediate psychological effects of COVID-19 upon nonmedical Chinese college students induced more severe anxiety and depression than those on medical students. Combining with previous reports showing that the impact of some public health events on college students or the general public is lasting without timely psychological intervention (34, 35), we believe that the mental health problems related to the COVID-19 outbreak among college students may persist and be amplified over time. Accordingly, we propose that long-term follow-ups and therapies involving professional psychological interventions and guidance should be promptly initiated, for these nonmedical students suffering during the COVID-19 outbreak, and the sooner the better.

Our study also identified potential risk factors for the development of depression, anxiety, and insomnia in these nonmedical vs. medical students. Undoubtedly, these risk factors might endure allostatic overload and promote the development of psychopathology, including chronic insomnia (36). Independent factors, i.e., being female or native of non-Shandong, were common risk factors for depression and anxiety among nonmedical students, while living in rural areas and age were risk factors of depression for medical students. Among these nonmedical students, females might be more sensitive to stress related to COVID-19. Previous researches have revealed that women were more vulnerable to stress- and fear-based disorders, such as anxiety and post-traumatic stress disorder (37). With regard to native of non-Shandong as another risk factor for these nonmedical students, we believe that the work of the local government and University personnel in Shandong was productive in the early stage of COVID-19 epidemic. Notably, with the initial COVID-19 outbreak, the Shandong government assisted local people in taking various measures to control the spread of the epidemic, including timely and transparent disclosures regarding data, popularization of knowledge about the spread and protection of COVID-19, and provisions of telemedicine (38). All of these efforts, predominantly in Shandong, would contribute to easing the burden of the epidemic within the local public, including nonmedical students. Among these medical students, it seems reasonable that the depression of living in rural areas was closely associated with the relative scarcity of medical resources in remote mountainous areas, as they were more aware of the importance of preventive measures against the epidemic (39). Moreover, the older medical students interning in hospital settings would inevitably face a higher risk of exposure to COVID-19 patients, thereby creating more psychological problems. In this way, nonmedical students with high risks of contact with COVID-19 patients in the community would experience depression like that of medical students.

In all, appropriate learning conditions (40) and psychological recovery programs (41) appear to be necessary. Primarily, those programs favor activities that contribute to optimal physical and mental health. Active participation may help nonmedical students quickly adapt to the distress caused by the COVID-19 epidemic and progress to optimal health, thereby achieving academic excellence. In addition, an enhanced dissemination of recent medical findings related to diagnosis, treatment, and prevention of COVID-19 (42) can serve as a means to shield nonmedical college students from distress of uncertainty about the unknown during the current global pandemic of COVID-19. In this regard, new media and short videos may be of great help in distributions of information (43). Encouragingly, the inception of a free, full coverage COVID-19 vaccinations nationwide in January 2021 should maximize the protection of these college students in China.

### Limitations

This present study has several limitations. First, as a cross-sectional design was used, the inadequacies associated with such a design cannot be avoided. A survey of the psychological changes of students in different periods of the COVID-19 epidemic would have provided a better understanding of the mental impact of the disease. Second, the data were acquired from psychological self-assessments via an online survey, which is bound to be some deviations. Therefore, further studies were encouraged to use clinical interviews for more comprehensive assessment. Third, as these medical and nonmedical students were not sampled independently, this may introduce some bias in the sampling procedure. Finally, the voluntary nature of the survey made follow-up difficult as very few students were willing to fill in the questionnaire again.

## Conclusion

In conclusion, a higher prevalence of psychological symptoms was present in nonmedical vs. medical students in universities within the Shandong Province during the COVID-19 epidemic recovery period, as well as associated risk factors. Nonmedical students would benefit from positive approaches, including supplemental learnings regarding comprehensive knowledge of COVID-19, recovery programs, and long-term psychological interventions to improve their psychological well-being.

## Data Availability Statement

The datasets presented in this article are not readily available due to participants' names and contact information. Requests to access the datasets should be directed to nuancheng471227@163.com.

## Ethics Statement

All participants provided their online informed consent. The study was approved by the Medical Ethics Committee of the Second Hospital of Shandong University [No. KYLL-2020(LW)-064]. All subjects were informed about the purpose of the study in accordance with Chinese legislation.

## Author Contributions

PW: conception and design. XZ, YG, HY, BY, HX, ZX, QL, JS, JB, WM, and PW: conduction. XZ and LL: statistical analysis. XZ, BY, HX, ZX, JS, and WM: administrative, technical, or material support. XZ: drafting of the manuscript. WM and PW: critical revision of the manuscript for important intellectual content. All authors read and approved the final paper.

## Conflict of Interest

The authors declare that the research was conducted in the absence of any commercial or financial relationships that could be construed as a potential conflict of interest.
